# Immediate three-dimensional changes in the oropharynx after different mandibular advancements in counterclockwise rotation orthognathic planning

**DOI:** 10.4317/jced.57913

**Published:** 2021-04-01

**Authors:** Caio-Bellini Lovisi, Neuza-Maria-Souza-Picorelli Assis, Daniel-Amaral-Alves Marlière, Karina-Lopes Devito, Fábio-Gamboa Ritto, Paulo-José-D’Albuquerque Medeiros, Bruno-Salles Sotto-Maior

**Affiliations:** 1 Department of Dental Clinic of Federal University of Juiz de Fora, Juiz de Fora, Minas Gerais, Brazil; 2 Division of Oral and Maxillofacial Surgery, Piracicaba Dental School, State University of Campinas, Piracicaba, São Paulo, Brazil; 3 Division of Oral and Maxillofacial Surgery, University Hospital Pedro Ernesto, State University of Rio de Janeiro, Rio de Janeiro, Brazil

## Abstract

**Background:**

A retrospective cohort study was performed to evaluate the immediate effect on the oropharynx dimensions from different mandibular advancements in patients undergone counterclockwise rotation (CCW) of the maxillomandibular complex.

**Material and Methods:**

138 CBCT images of patients, who had undergone orthognathic surgery, were identified from Dolphin Imaging archive according to pre- (T0) and post-operative (T1) times. Each pre-operative CBCT image was selected considering retrognathic mandible. Superimpositions of CBCT images were performed to measure mandibular advancement at B point in millimeters (mm) and divided into three groups: G1 (< 5 mm), G2 (between 5 and 10 mm) and G3 (> 10 mm). For evaluating oropharynx dimension at T0 and T1 for each group, medial sagittal area (MSA), volume, and minimum cross-sectional axial area (CSA) were measured on Dolphin Imaging. Pearson correlation verified reliability of method. Paired t-test were applied to compare values of measurements between T0 and T1 (*p* ≤ 0.05).

**Results:**

88 CBCT images were included. Method was reliable (r ≥ 0.93). According to MSA, volume and CSA values from G1, there was no significant difference between T0 and T1. CSA values presented significant difference comparing T0 and T1 in G2 (*p* ≤ 0.05). In subjects of G3, measurements increased in T1 significantly affecting oropharynx dimension.

**Conclusions:**

MSA, volume and CSA values showed a significant increase affecting upper airway in advancements higher than 10 mm. Mandibular advancement range showed different effects in the airway space and should be considered to achieve favorable post-operative results in the oropharynx dimensions.

** Key words:**Retrognathia, orthognathic surgery, three-dimensional imaging, oropharynx, airway.

## Introduction

Class II dentofacial deformities or mandibular retrognathic patients tend to show a narrow oropharynx dimensions or upper airway (UA). Other factors commonly found in class II patients, such as increased vertical length of the UA, high occlusal plane and retrusion of pogonion may increase airflow resistance (1). Decreased airway space and increased resistance to airflow may lead to a severe narrowing or to a transitory obstruction in the minimal axial area resulting in one of the predisposing factors for obstructive sleep apnea (2-3).

Orthognathic surgery is performed to correct bone deformities and facial soft tissue discrepancies (4). Commonly, bimaxillary advancements cause major skeleton modifications increasing UA area and volume (3). As usual, bimaxillary advancements equal to or higher than 10 millimeters (mm) were reported as favorable changes to the UA dimensions (3,5-7). In this sense, mandibular movements seem to be more important than maxillary advancement (8). Since mandibular advancement stretches the pharynx and the suprahyoid muscles, airway gain may be enhanced with a counterclockwise (CCW) maxillomandibular rotation. The occlusal plane rotation was able to provide pogonion and B point move forward farther than the lower teeth maximizing the advancement of the hyoid bone, base of the tongue, genioglossus, and geniohyoid muscles (9).

 Traditionally, lateral cephalograms have been used to evaluate airway parameters (10). However, bidimensional images have presented limitations for evaluating a three-dimensional structure (11-13). Three-dimensional images can be used to reconstruct and evaluate airway spaces from computed tomography scans (1-2,5-6). Furthermore, cone beam computed tomography (CBCT) has shown an appropriated image method to verify UA dimensions in patients undergone orthognathic surgery (8,14-15).

Three-dimensional assessments were developed to measure UA changes using CBCT images to compare pre- and postoperative of CCW rotation in a patient sample of maxillomandibular advancements higher than 10 mm (16-18). However, if their single groups of mandibular advancement were only considered to CCW rotation and guaranteed a significant increase of the UA dimensions, this magnitude of surgical movement could not aesthetically be accepTable planning to all patients. Therefore, our aim was to evaluate the immediate effect on the oropharynx dimensions from different mandibular advancements in patients undergone Orthognathic surgery by CCW maxillomandibular rotation planning.

## Material and Methods

This retrospective cohort study was performed by using 138 pre- and postoperative CBCT scans of patients who had undergone orthognathic surgery at the University Hospital of Pedro Ernesto, State University of Rio de Janeiro (Rio de Janeiro, Brazil) between January 2012 and January 2016.

All subjects were scanned in the same I-CAT scanner (Imaging Sciences International, Hatfield, Pennsylvania, USA), operating at 120 kV, 5 mA, FOV of 22 x 13 cm, isotropic voxel of 0.3 mm, and 14-bit grey scale. The CBCT scans were taken according to previous protocol developed at our Oral and Maxillofacial Surgery Division, patients were instructed to sit upright with a natural head position and asked to breathe slowly and not to swallow. The mandible was positioned in a centric relation with manual manipulation and no use of interocclusal device (15). For all CBCT acquisitions, two time points were considered pre-operative (T0), and immediately up to 15 days after surgery (T2). The DICOM images were imported and archived into Dolphin Imaging 11.7 (Dolphin Imaging and Management Solutions, Chatsworth, Calif., USA), which was carried out the same workflow of orthognathic surgery planning. All orthognathic surgeries were conducted by the same Oral and Maxillofacial Surgeon team. The study was approved by the Research Ethics Committee of the Federal University of Juiz de Fora and State University of Rio de Janeiro regarding the use of data and performed according to the ethical principles and Declaration of Helsinki.

From each patient and clinical records, subjects were selected according to the following inclusion criteria: (I) availability of pre- and post-operative CBCT data imported into Dolphin Imaging software; (II) patients with retrognathic mandible from Steiner’s cephalometric analysis (angle between Sella point-nasion-B point, SNB < 78º); (III) bi-maxillary orthognathic surgery by CCW maxillomandibular complex rotation as planned. Exclusion criteria were: (I) patients with asymmetric mandible; (II) growing patients (III) history of adjuvant surgery in the soft tissues of the head and neck region; (IV) trans-surgical or post-operative complications; and (V) incomplete records.

-Cranial base superimposition and B point measurements in mandibular advancements

From subjects available at Dolphin Imaging software, three-dimensional soft and hard tissue had been segmented from each pre-operative DICOM image and patient’s heads were positioned in an estimated natural position (15) before performing planning workflow. Thereupon, each post-operative DICOM image was superimposed over pre-operative CBCT volume by an operator (C.B.L), who used Superimpose Tool. Axial, sagittal, and coronal slices of the CBCT volumes were used to select the anatomical structures of the skull base supporting alignment between post-operative CBCT images in relation to pre-operative one by using a voxel-based superimposition. This superimposition method was used to keep on the same pre- and post-operative head position considering cranial base with no changes after surgical procedures (19-20).

**Figure 1:**
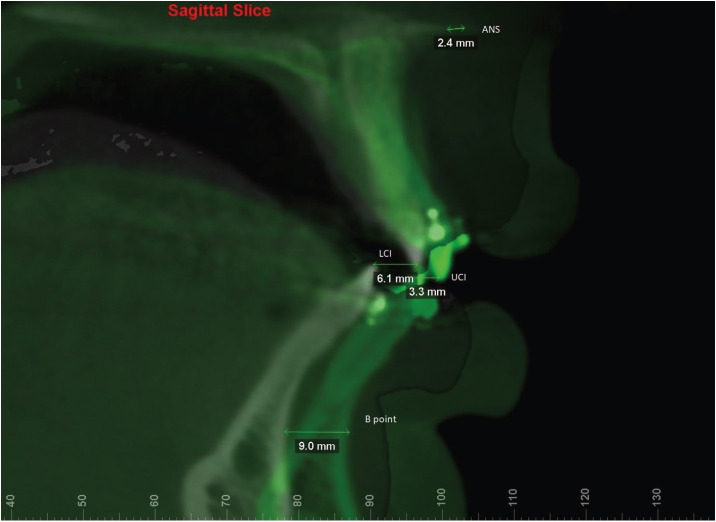
Superimposition of T0 and T1 and measurements of the advancements at anterior nasal spine (ANS), upper central incisor (UCI), lower central incisor (LCI) and B point.

After performing the superimpositions, the same operator (C.B.L) selected Measure tool following as reference the sagittal plane at Dolphin Imaging software to determine linear measurement of the anterior nasal spine (ANS), upper central incisor (UCI), lower central incisor (LCI), and B point. From linear measurements in mm, CCW maxillomandibular rotations were confirmed by verifying comparisons between linear measurements either B points were higher than LCI or UCI advanced more than ANS (Fig. 1). According to mandibular advancement measurements in B point, subjects were allocated to three groups: G1 (advancement < 5 mm), G2 (advancement between 5 and 10 mm) and G3 (advancement > 10 mm).

-Oropharynx dimensions evaluation

The Sinus/Airway Evaluation Tool in the Dolphin Imaging software was used for reconstruction and evaluation of the oropharynx (12,15-18,21). At First, the oropharynx anatomical references were delimited in the medial sagittal reconstruction and described such as: anterior, lateral and posterior limits were defined by soft tissue contour of pharyngeal walls; upper limit, retropalatal region delimited by a parallel line to the horizontal plane from posterior nasal spine extending to the posterior wall of the pharynx; lower limit, a parallel line to the horizontal plane crossing at the height of the base of the epiglottis to posterior wall of hypopharynx.

**Figure 2:**
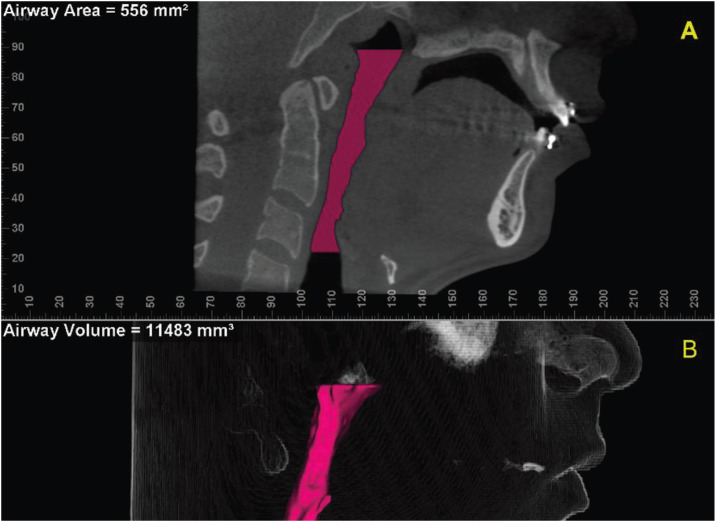
Oropharynx delimited in the CBCT: (A) Sagittal reconstruction indicating the medial sagittal area (MSA); (B) Three-dimensional reconstruction indicating the volume of oropharynx.

**Figure 3:**
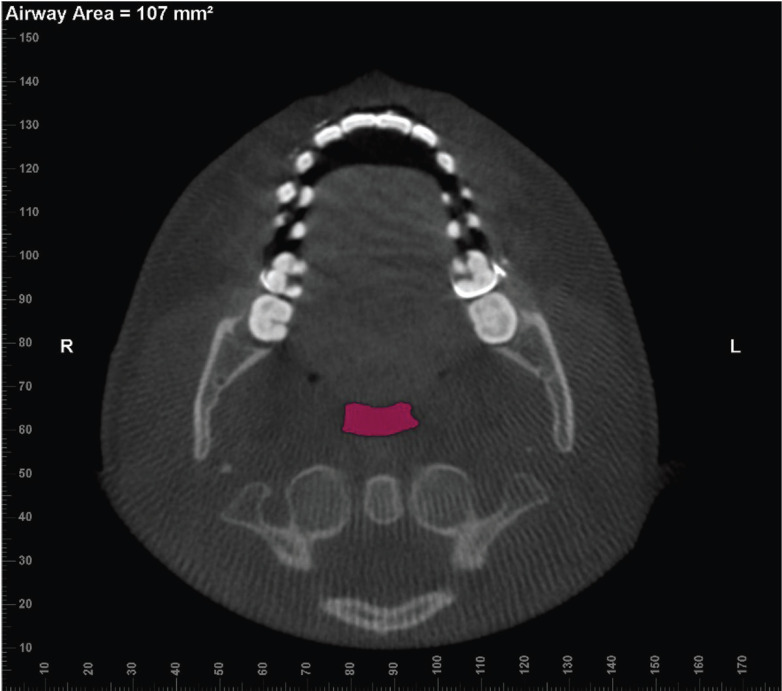
Axial reconstruction of CBCT image indicating the minimum cross-sectional axial area (CSA).

Next, the Add Seed Points Tool was used to insert seed points inside this area. The detection sensitivity of the airway space was standardized at 25%, and the Update Volume Tool was used to calculate the medial sagittal area in mm2 (MSA) and the Volume in the airway space of oropharynx in mm3, as previously delimited (Fig. 2) (13). The minimum cross-sectional axial area in mm2 (CSA) was measured using the option Enable Minimum Axial Area in the axial view (Fig. 3). All analysis was performed by the same evaluator (C.B.L) limiting a maximum of 5 subject CBCT images at 1-week intervals. All workflow of oropharynx dimension evaluations was measured twice considering abovementioned interval of times.

-Statistical analysis

Statistical analysis was performed with SPSS software (Statistics IBM software version 15.0; IBM Corp, Armonk, NY). Pearson’s correlation coefficients (r) were calculated to assess reliability of the intra-rater method.

Descriptive analysis was able to show the minimum, maximum, means and standard deviation (SD) for linear measurements ANS, UCI, LCI and B point between pre- and post-operative images superimposed, according to G1, G2 and G3. Paired t-tests were used to compare oropharynx dimensions (MSA, volume and minimal CSA) between T0 and T1 in each group (G1, G2 and G3). Statistical significance was set at *p* ≤ 0.05.

## Results

From 138 CBCT pre- and post-operative images assessed, 88 CBCT images were selected according to inclusion criteria. Hence, forty-four skeletal class II patients were analyzed for the study sample, 33 females and 11 males, and range in age from 18 to 40 years.

Pearson’s correlation coefficient was excellent (r ≥ 0.93) showing intra-rater agreement, and the method was reliable. The patients were assigned to 13 subjects in G1 (B point measurement < 5 mm), 19 in G2 (5 mm < B point measurement < 10 mm), and 12 in G3 (B point measurement ≥ 10 mm). Table 1 presents mean B point measurement and descriptive results of linear measurements between others cephalometric references from pre- and postoperative CBCT image superimpositions.

**Table 1:**
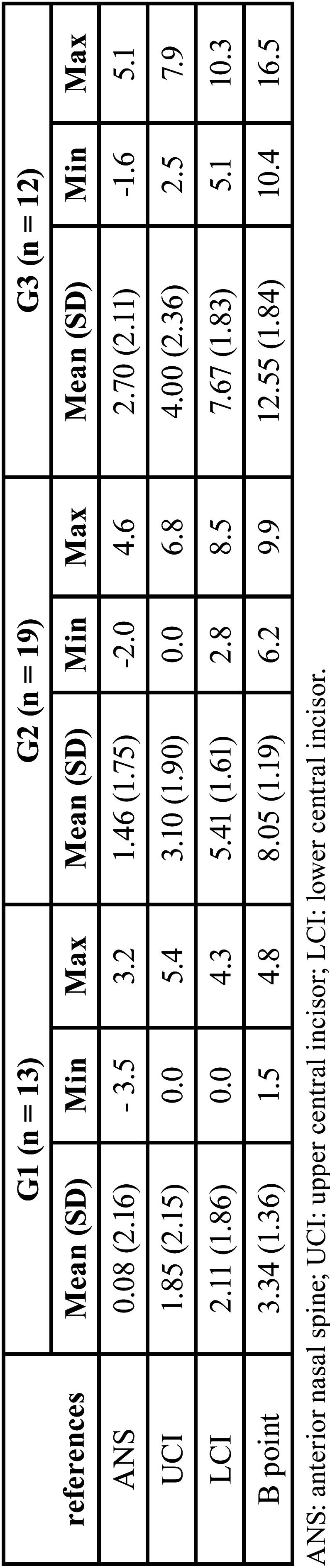
Mean, standard deviation, minimum and maximum values of the linear measurements between references from CBCT images superimpositions.

Table 2 presents Paired t-test results, which were statistically significant (*p* ≤ 0.05) for G3 when values of the MSA, volume and CSA were compared between pre-(T0) and postoperative (T1). For G3 subjects, Table 2 shows that there was a mean higher than 20% increase in the oropharynx dimension considering MSA, Volume and CSA measured.

**Table 2:**
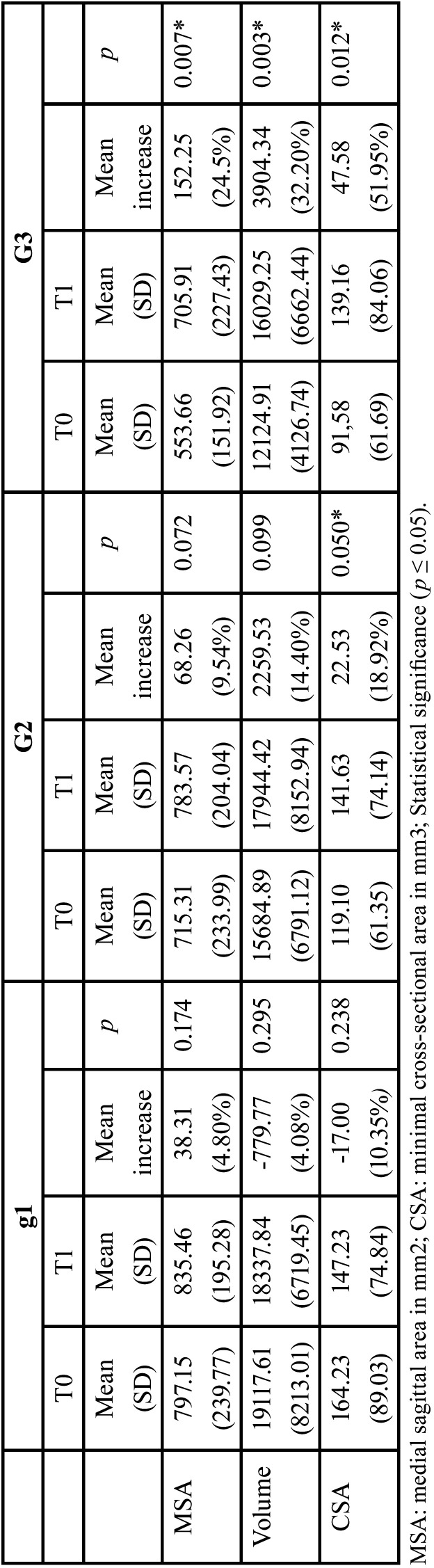
Paired t-test results compared the means of each variable of the oropharynx dimension measured between T0 and T1 for each group.

## Discussion

Oropharynx dimensions has been selected for assessment in retrognathic patients since they can present constriction areas in the UA (6). Afterward orthognathic surgery, mandibular movements can be more susceptible to provide effects in the oropharynx region than nasopharynx and hypopharynx (1,5,13). And, mandibular advancements presented more favorable impact than maxillary movements in the oropharynx dimensions (8). Hence, our method categorized each subject (pre-operative CBCT image) regarding at different mandibular advancement (G1, G2 and G3), and then, evaluated the effects in the oropharynx dimension comparing pre- (T0) and postoperative (T1) CBCT images.

From assessment of UA comparing between lateral cephalometric radiographs and CBCT images, Aboudara *et al.* (2009) established that CBCT scan is a simple and effective method to provide an accurately analysis of the airway area and volumetric measurement. As CBCT segmentations in software became possible, several methods have been developed to measure UA after orthognathic surgery, providing the accurate morphology by means of the measurements of MSA, minimal CSA, and volume (1). Commonly, volume and minimal CSA were considered the most important parameters for showing the total gain capacity of the oropharynx dimension (1,6,22). Our results indicated higher effects in the oropharynx dimensions after CCW maxillomandibular complex rotation from increasing the volume and minimal CSA measurements means, regardless group or statistically significant difference.

In accordance with each category of the subjects (G1, G2 and G3), mandibular advancements lower than 5 mm (G1) did not produce significant changes in the oropharynx dimensions in any of the variables. The lack of change may be related to the small amount of advancement, which may have been insufficient to stretch the suprahyoid musculature (17). Nevertheless, a significant increase in UA space with similar advancements has been reported. Ristow *et al.* (2018) found a significant difference in oropharynx volume and minimal CSA, with a mean value of mandibular advancement of 4.77 mm measured at three different points (left and right mental foramen and pogonion). These authors used two different programs to evaluate mandibular advancement and the airways, instead of using Dolphin Imaging. Hence, different software for assessing UA may generate differences among the results (24).

Previous studies have presented significant improvements in minimal CSA and volume with mandibular advancements between 5 and 10 mm (13,17,21). This is partially in accordance with the present study since G2 presented significant improvements in minimal CSA, but no statistically significant difference was observed when the oropharynx volume was evaluated. This difference in volume can be explained by stipulating different anatomical limits for oropharynx, patient position during computerized tomography acquisition, planning of different surgical advancements, and the time elapsed for evaluation. Based on method of anatomical delimits, Brunetto *et al.* (2014) included part of the nasopharynx in their analysis and performed higher maxillary advancements (mean 4.71 mm), whereas our method was restricted to oropharynx and mean of maxillary advancement was 1.46 mm at ANS and 3.10 mm at UCI (G2). In addition to using different anatomical limits, Kochar *et al.* (2016) evaluated the oropharynx dimensions after isolated mandibular surgeries by using multislice computerized tomography. Hence, differences between results may be related to patient position during scans images because our protocol of CBCT scans were performed in sitting position, whereas supine position in computerized tomography. Thus, we suppose that patient position may alter the UA due to the gravitational forces that displace the tongue and soft palate posteriorly (25). Kochar *et al.* (2016) and Brunetto *et al.* (2014) evaluated the UA at least 5 months of postoperatively, which may be different from an evaluation conducted during the immediate postoperative period (12).

Advancements higher than 10 mm are commonly related to an enlargement of the airways with linear bimaxillary advancement (3,5-7). However, these linear maxillary advancements are not always possible from an esthetic point of view, creating a biprotuse profile with an acute naso-labial angle (1). Thus, the CCW rotation, in addition to improving the airway, as shown in the present study (G3), enhances the esthetic profile of class II patients by optimizing the advancement of the pogonion and avoiding the unpleasant protrusion of the maxilla in the patient (4).

CCW rotation with mandibular advancements higher than 10 mm has been related to significant increases in MSA, volume and minimal CSA in the oropharynx (1,12,16,18). Raffaini and Pisani (2012) evaluated 10 patients with mandibular advancements ranging from 10 to 18 mm and showed gains in the oropharynx dimension of 34% in MSA, 56% in volume, and 112% in minimal CSA. Besides finding gains of 178 mm2 in surface area, 10.118 mm3 in volume, and 76.67 mm2 in CSA, Miranda *et al.* (2015) evaluated 23 patients with a mean advancement of 14 mm and did not report the sensitivity parameter of the airway used on Dolphin Imaging. Comparatively with our results, their values detected higher impact in the oropharynx dimensions because it may be related to sample size, amount of mandibular advancement, assessment of method, patient’s overweight, and individual differences in muscle tone around the pharyngeal airways (26).

Despite the present study did not consider long-term postoperative outcomes, previous study reported long-term stability of the skeletal movement after counterclockwise rotation using rigid fixation (27). And long-term stability of oropharyngeal airway space has also kept on sTable after postsurgical follow-up of 34 months (28). On the other hand, a long-term follow-up of the maintenance of the dimension of the UA after CCW rotation would require a strict control of all of the different variables which may predispose changes, such as an increase of the body mass index, muscle tone analysis, connective tissue flaccidity, and adipose tissue distribution (15).

There was limitation of our study because some important clinical evaluation should have been considered, such as body mass index, the Berlin questionnaire, the Epworth Sleepiness Scale, and polysomnography. And, we recognize that prospective study including different CCW advancements and evaluating three-dimensional changes with a strict control of the external factors that influences the changes in the UA would be conducted.

## Conclusions

In subjects with mandibular advancements between 5 and 10 mm, only minimal CSA was significantly affected from comparing pre- and post-operative. Values of MSA, volume and CSA showed a significant increase in the oropharynx dimension of the subjects with mandibular advancements higher than 10 mm. Therefore, range of mandibular advancements showed different effects in the upper airway space, and it should be considered in CCW rotation planning to look forward to favorable post-operative results in the oropharynx.

## References

[1] Zinser MJ, Zachowb S, Sailer HF (2013). Bimaxillary 'rotation advancement' procedures in patients with obstructive sleep apnea: a 3-dimensional airway analysis of morphological changes. Int J Oral Maxillofac Surg.

[2] Li KK (2011). Maxillomandibular advancement for obstructive sleep apnea. J Oral Maxillofac Surg.

[3] Bianchi A, Betti E, Tarsitano A, Labate AMM, Lancellotti L, Marchetti C (2014). Volumetric three-dimensional computed tomographic evaluation of the upper airway in patients with obstructive sleep apnoea syndrome treated by maxillomandibular advancement. Br J Oral Maxillofac Surg.

[4] Louro RS, Calasans-maia JA, Mattos CT, Masterson D, Calasans-Maia MD, Maia LC (2018). Three-dimensional changes to the upper airway after maxillomandibular advancement with counterclockwise rotation: a systematic review and meta-analysis. Int J Oral Maxillofac Surg.

[5] Fairburn SC, Waite PD, Vilos G, Harding SM, Bernreuter W, Cure J (2005). Three-dimensional changes in upper airways of patients with obstructive sleep apnea following maxillomandibular advancement. J Oral Maxillofac Surg.

[6] Abramson Z, Susarla MS, Lawler M, Bouchard C, Troulis M, Kaban LB (2011). Three-dimensional computed tomographic airway analysis of patients with obstructive sleep apnea treated by maxillomandibular advancement. J Oral Maxillofac Surg.

[7] Boyd SB, Walters AS, Song Y, Wang L (2013). Comparative effectiveness of maxillomandibular advancement and uvulopalatopharyngoplasty for the treatment of moderate to severe obstructive sleep apnea. J Oral Maxillofac Surg.

[8] Hernández-Alfaro F, Martínez RG, Bueno JM (2011). Effect of mono and bimaxillary advancement on pharyngeal airway volume: cone-beam computed tomography evaluation. J Oral Maxillofac Surg.

[9] Mehra P, Downie M, Pita MC, Wolford LM (2001). Pharyngeal airway space changes after counterclockwise rotation of the maxillomandibular complex. Am J Orthod Dentofacial Orthop.

[10] Sahoo NK, Jayan B, Ramakirshna N, Chopra SS, Kochar G (2012). Evaluation of upper airway dimensional changes and hyoid position following mandibular advancement in patients with skeletal class II malocclusion. J Craniomaxillofac Surg.

[11] Aboudara C, Nielsen I, Huang JC, Maki K, Miller AJ, Hatcher D (2009). Comparison of airway space with conventional lateral headfilms and 3-dimensional reconstruction from cone-beam computed tomography. Am J Orthod Dentofacial Orthop.

[12] Carvalho ACG, Filho OM, Garcia Jr IR, Araujo PM, Nogueira RLM (2012). Cephalometric and three-dimensional assessment of superior posterior airway space after maxillomandibular advancement. Int J Oral Maxillofac Surg.

[13] Kochar GD, Chakranarayan A, Kohli S, Kohli VS, Khanna V, Jayan B (2016). Effect of surgical mandibular advancement on pharyngeal airway dimensions: a three-dimensional computed tomography study. Int J Oral Maxillofac Surg.

[14] Tso HH, Lee JS, Huang JC, Maki K, Hatcher D, Miller AJ (2009). Evaluation of the human airway using cone-beam computerized tomography. Oral Surg Oral Med Oral Pathol Oral Radiol Endod.

[15] Canellas JV, Barros HLM, Medeiros PJD, Ritto FG (2016). Effects of surgical correction of class III malocclusion on the pharyngeal airway and its influence on sleep apnea. Int J Oral Maxillofac Surg.

[16] Raffaini M, Pisani C (2012). Clinical and cone-beam computed tomography evaluation of the three-dimensional increase in pharyngeal airway space following maxillo-mandibular rotation-advancement for Class II-correction in patients without sleep apnoea (OSA). J Craniomaxillofac Surg.

[17] Gonçalves JR, Gomes LCR, Vianna AP, Rodrigues DB, Gonçalves DAG, Wolford LM (2013). Airway space changes after maxillomandibular counterclockwise rotation and mandibular advancement with TMJ Concepts total joint prostheses: three-dimensional assessment. Int J Oral Maxillofac Surg.

[18] Miranda WS, Rocha VAC, Marques KLS, Neto AIT, Prado CJ, Zanetta-barbosa D (2015). Three-dimensional evaluation of superior airway space after orthognathic surgery with counterclockwise rotation and advancement of the maxillomandibular complex in Class II patients. Oral Surg Oral Med Oral Pathol Oral Radiol.

[19] Ritto FG, Schmitt ARM, Pimentel T, Canellas JV, Medeiros PJ (2017). Comparison of the accuracy of maxillary position between conventional model surgery and virtual surgical planning. Int J Oral and Maxillofac Surg.

[20] Marlière DAA, Demétrio MS, Verner FS, Asprino L, Chaves Netto HDM (2019). Feasibility of iterative closest point algorithm for accuracy between virtual surgical planning and orthognathic surgery outcomes. J Craniomaxillofac Surg.

[21] Brunetto DP, Velasco L, Koerich L, Araújo MTS (2014). Prediction of 3-dimensional pharyngeal airway changes after orthognathic surgery: A preliminary study. Am J Orthod Dentofacial Orthop.

[22] Schendel SA, Broujerdi JA, Jacobsonc RL (2014). Three-dimensional upper-airway changes with maxillomandibular advancement for obstructive sleep apnea treatment. Am J Orthod Dentofacial Orthop.

[23] Ristow O, Rückschloß T, Berger M, Grotz T, Kargus S, Krisam J (2018). Short- and long-term changes of the pharyngeal airway after surgical mandibular advancement in class II patientsda three-dimensional retrospective study. J Craniomaxillofac Surg.

[24] El H, Palomo JM (2010). Measuring the airway in 3 dimensions: A reliability and accuracy study. Am J Orthod Dentofacial Orthop.

[25] Kim MA, Kim BR, Young JK, Kim YJR, Park YH (2014). Head posture and pharyngeal airway volume changes after bimaxillary surgery for mandibular prognathism. J Craniomaxillofac Surg.

[26] Kim JS, Kim JK, Hong SC, Cho JH (2010). Pharyngeal airway changes after sagittal split ramus osteotomy of the mandible: a comparison between genders. J Oral Maxillofac Surg.

[27] Chemello PD, Wolford LM, Buschang PH (1996). Occlusal plane alteration in orthognathic surgery-part II: Long-term stability of results. Am J Orthod Dentofacial Orthop.

[28] Gonçalves JR, Buschang PH, Goncalves DG, Wolford LM (2006). Postsurgical stability of oropharyngeal airway changes following counter-clockwise maxillo-mandibular advancement surgery. J Oral Maxillofac Surg.

